# Supplementary material for: Aryl Hydrocarbon Receptor-Dependent and -Independent Pathways Mediate Curcumin Anti-Aging Effects
Source: Antioxidants (Basel). 2022 Mar 23;11(4):613. doi: 10.3390/antiox11040613 (PMC9024831; doi:10.3390/antiox11040613)
Supplement: Supplementary file 1 [file antioxidants-11-00613-s001.zip › antioxidants-1638200-supplementary.pdf]

Article

Aryl Hydrocarbon Receptor-Dependent and -Independent Pathways Mediate Curcumin Anti-Aging Effects

Vanessa Brinkmann [1,2,†], Margherita Romeo [1,2,†], Lucie Larigot [3], Anne Hemmers [2], Lisa Tschage [2], Jennifer Kleinjohann [2], Alfonso Schiavi [1,2], Swantje Steinwachs [2], Charlotte Esser [2], Ralph Menzel [4], Sara Giani Tagliabue [5], Laura Bonati [5], Fiona Cox [1,6], Niloofar Ale-Agha [1], Philipp Jakobs [1], Joachim Altschmied [1,2], Judith Haendeler [1], Xavier Coumoul [3] and Natascia Ventura [1,2,*]

1 Institute of Clinical Chemistry and Laboratory Diagnostic, Medical Faculty, Heinrich Heine University, Düsseldorf, Moorenstr 5, 40225 Düsseldorf, Germany; vanessa.brinkmann@uni-duesseldorf.de (V.B.); margherita.romeo@iuf-duesseldorf.de (M.R.); alfonso.schiavi@uni-duesseldorf.de (A.S.); ficox100@uni-duesseldorf.de (F.C.); Niloofar.ALE-AGHA@uni-duesseldorf.de (N.A.-A.); philipp.jakobs@hhu.de (P.J.); joalt001@hhu.de (J.A.); juhae001@hhu.de (J.H.)

2 IUF – Leibniz Research Institute for Environmental Medicine, Auf'm Hennekamp 50, 40225 Düsseldorf, Germany; anne.hemmers@hhu.de (A.H.); tschage@em.uni-frankfurt.de (L.T.); jenny.kleinjohann@web.de (J.K.); swantje.steinwachs@iuf-duesseldorf.de (S.S.); charlotte.esser@uni-duesseldorf.de (C.E.)

3 Faculté des Sciences Fondamentales et Biomédicales, Université de Paris, 45 rue des Saints-Pères, F-75006 Paris, France; luciole271@gmail.com (L.L.); xavier.coumoul@parisdescartes.fr (X.C.)

4 Institute of Biology, Humboldt-University Berlin, Philippstr. 13, 10115 Berlin, Germany; ralph.menzel@biologie.hu-berlin.de

5 Department of Earth and Environmental Sciences, University of Milano-Bicocca, Piazza della Scienza 1, 20126 Milano, Italy; s.gianitagliabue@gmail.com (S.G.T.); laura.bonati@unimib.it (L.B.)

6 Institute of Clinical Pharmacology and Pharmacology, Medical Faculty, University Hospital and Heinrich Heine University, Düsseldorf, Moorenstr 5, 40225 Düsseldorf, Germany

* Correspondence: natascia.ventura@uni-duesseldorf.de, +49-211-3389203

† These authors contributed equally to this work.

Brinkmann et. al

Supplementary figures and tables

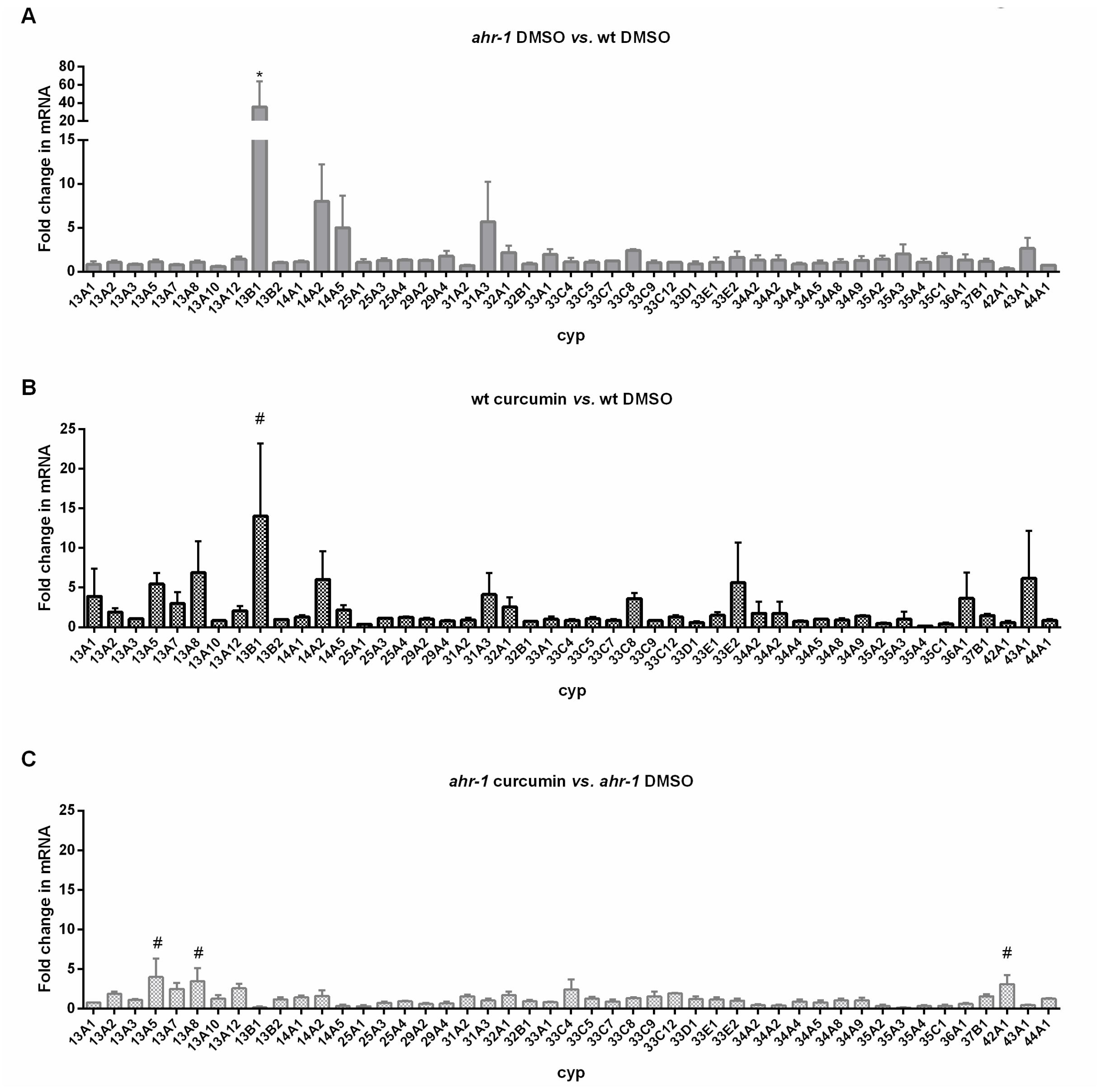

Figure S1. *Cyp-13B1* expression is increased by curcumin in an *ahr-1*-dependent manner. A) *cyp* gene expression in DMSO-treated *ahr-1(ju145)* relative to DMSO-treated wild-type. **B)** *cyp* gene expression in curcumin-treated wild-type relative to DMSO-treated wild-type. **C)** *cyp* gene expression in curcumin-treated *ahr-1* mutants relative to DMSO-treated *ahr-1* mutants. Mean + SEM of pooled data from 3 replicates is shown in all panels. * p-value < 0.05 *vs.* wt, $^{\#}$ p-value < 0.05 *vs.* DMSO, statistical test: 2-way ANOVA with Tukey's multiple comparisons test.

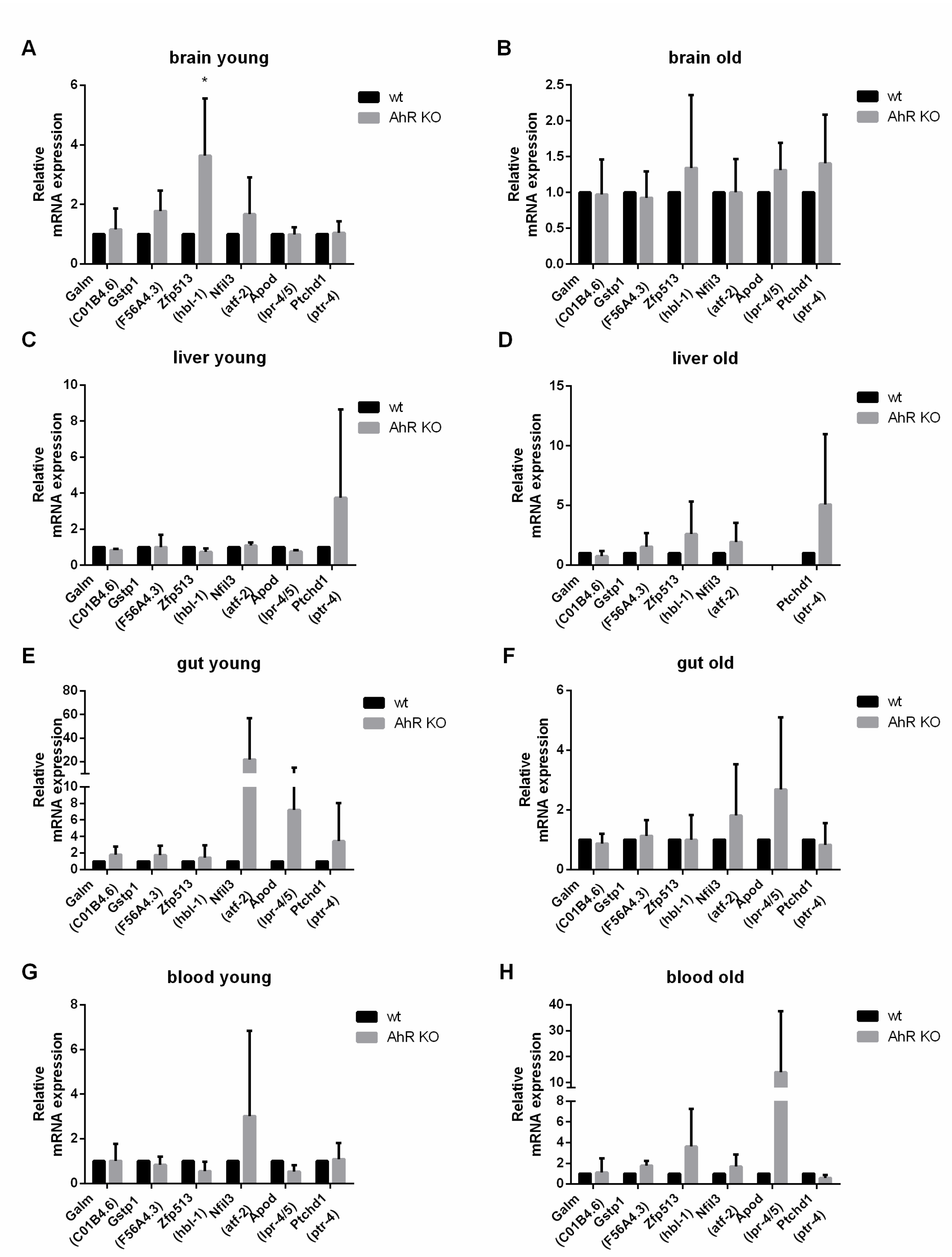

Figure S2. Tissue-specific gene expression changes in mice. Expression levels of homologs of some of the strongest differentially expressed genes between wt and *ahr-1 C. elegans* in the brain **(A-B)**, liver **(C-D)**, gut **(E-F)**, and blood **(G-H)** of young (8-12 weeks old) or old (18 months old) mice was assessed by qPCR. The *C. elegans* homolog names are indicated in brackets. Mean + SD of 3 mice is shown. Statistical test: 2-way ANOVA with Sidak's multiple comparisons test, * p-value < 0.05 vs. wt.

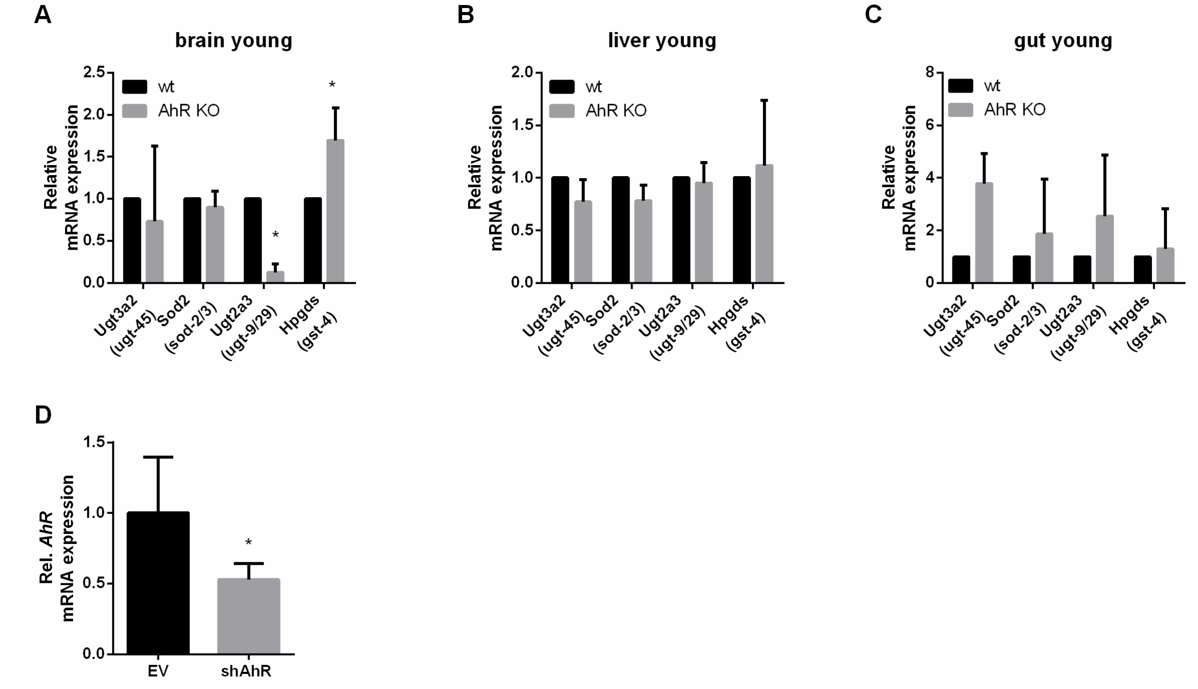

Figure S3. Genes coding for detoxification enzymes are differentially expressed in a tissue-specific manner in mice. qPCR analysis of Ugt3A2, Sod2, Ugt2A3, and Hpdgs expression in the brain (A), liver (B), and gut (C) young (8-12 weeks old) or old (18 months old) mice. Mean + SD of pooled data from 3 mice are shown. Statistical test: 2-way ANOVA with Sidak's multiple comparisons test, * p-value < 0.05 vs. wt. D) Human primary EC were transfected with an empty vector (EV) or an expression vector for an shRNA targeting the human AhR transcript (shAhR). Relative *AhR* expression was assessed by qPCR, mean expression in the EV transfected cells was set to 1. Data are shown as mean + SD from 7 experiments. [#]p<0.05 vs. EV.

Table S1. Primer pairs used for qPCR with *C. elegans* samples. Gene names are sorted alphabetically.

Gene name	Forward Primer sequence (5′ – 3′)	Reverse Primer sequence (5′ – 3′)	Efficiency (%)[a]	RefSeq mRNA ID
**act-1**	GCTCTTGCCCCATCAACCAT	CACTTGCGGTGAACGATGGA	94.8-100.4	NM_073418
**atf-2**	CGAAGGAACAATGAAGCCGC	CCAAGAGCTGAACTCGTCGT	98.1	NM_063460
**C01B4.6**	TGGCGATGCGAAAATTGATGTAA	ATCTCCAGAAAGTGCTCGGC	83.9	NM_071283
**C01B4.7**	GTTTTGGAATCAGACGCGGG	CAGTGGGGTTCCGTCAAGTT	106.6	NM_071282
cdc-42	ATTACGCCGTCACAGTAATG	ATCCCTGAGATCGACTTGAG	80.8-107.2	NM_063197
clec-209	TGCTCGGGGAACAACCAAAA	TTGGCTACGAACGATTGATGC	88.4	NM_071249
cyp-13A1	GTACAACCTACACAAATCGC	TGAATCTCTGGATGAGTTGC	91.2	NM_001383923
cyp-13A2	GTGGACAAAGAAATATGGCAA	ACAAGTGAACTCGTTGTTCT	97.6	NM_063708
cyp-13A3	GCCTGAAAGATGGGAATCTG	TGCCAGAAGCATCTTTTCTT	100.5	NM_063709
cyp-13A5	TGGACAAAAGAATACGGACC	CGTAGGTGATGACAGAGTTC	86.7	NM_063711
cyp-13A7	GACAAAGAAATACGGACCTG	TGCAGTTAATTTTCTCCCGT	93.2	NM_063713
cyp-13A8	AGCTCAAGGTTTTAGATGGA	CATAAGCTCCTGTGTGCTAT	94	NM_063714
cyp-13A10	AAGACAGTGCGATGGAATTA	TCTCCGAATATCGCTCTGA	103.7	NM_063684
cyp-13A12	CTGGGAGCTTTAGCAAATTC	TCTCCTGATTCCCATCTTTC	105.8	NM_067304
cyp-13B1	GGGCTGTTCCAGTGTTAGTC	AACATCGTGAACCTGCTTTA	96.2	NM_077832
cyp-13B2	TGAGAATGTATCCAGTTGCC	GATTCGAGCCATCTTTCTGG	98.7	NM_077968
cyp-14A1	TTCACCAGTTCCCTCCAGAC	AGAACGGTGAGCTCGGTAAG	94.4	NM_077802
cyp-14A2	AGTCCGGAAGGAAATACTTG	CATCTCGGTTTGTATGAGTC	102.3	NM_077803
cyp-14A5	ATAGGCACGGCGAGACTACC	CAAGTACAGTCTTGTTCAAC	100.1	NM_072034
cyp-25A1	TGGCTCTCCTGATTTTAAGC	AAGTTTTTCCTTGCGTTCTG	84.7	NM_065374

cyp-25A2	CTCTTCTGATTCTCACCTCAA	TTTTTCTTTCCGGTCAACGA	93.1	NM_065375
cyp-25A3	AGCACTTCTTTCCTTTTCCC	TAAACATCTGCAAGTCCCTC	109.2	NM_001047385
cyp-25A4	CCAGTGTCTTTGATACTTCC	ACCCTCTCCAGCACGTCTTC	106.4	NM_065378
cyp-29A2	TGCCATAATTCTGGCATATC	AGGACCAGGTAGTTTACTTC	90.3	NM_073446
cyp-29A4	TTCCAGTGGCTCTTGCATTG	CATCTTTCCTCCA-TAAATCCAG	107.4	NM_073089
cyp-31A2	CCGCTGTACTTCTCGCTATG	TTCTTCATCTGCTCCGAGGC	84.3	NM_069751
cyp-31A3	TACTCCGCCGATCTCGTG	TTCTTCCTCTGCTCCGAGGG	81.8	NM_068236
cyp-32A1	TCCTAGCTGATGCAGTCGAG	CCGTTCTTGGATGACCTTTC	94.6	NM_072608
cyp-32B1	AGTTTTTGCATGTTCGGAAG	ACCATAACTTCAACACACCA	97.8	NM_071197
cyp-33A1	GAAGTTAATATTCAGGAACAT	TACGCGAAGTAGACACGAAG	100.6	NM_072587
cyp-33C4	AAAATCTTCCCAGACCCCT	GAAGACGGTGAGATCTT-GTATCTA	107.2	NM_071211
cyp-33C5	GTTTGTTTGTTCCTGTTCCA	CATATGGAATGTTGCCCATC	92.4	NM_071215
cyp-33C7	GAAGATTTGATGAAAGACTGGA	CAGAGGTCCAAGCAAGTATT	97.3	NM_071217
cyp-33C8	GATGATGTGCTCAACTACTG	CTTGAGCCTTTTGCTTCTTC	111.6	NM_071650
cyp-33C9	ATTTTCAAGCCGGAGAGATT	CGGAACAACCCTGTATCTAT	111	NM_071445
cyp-33C12	ATATTGTCCCGATCAACCAG	TGGGTCTGGGAAAACCTTAT	102.1	NM_071198
cyp-33D1	CGTTTTGCAACAAACAACTC	CCAATGACTCTGTCTAGCTC	106.1	NM_074675
cyp-33E1	CAATGCAACTGTTAATGAATCT	TCAGGGAATATCTCTGGATC	103.6	NM_069079
cyp-33E2	ATGAATCACAACGTCTTGCC	TCAGGGAAGATTTCTGGATT	100.2	NM_069069
cyp-34A2	TGTGCGTGTCTTCTCAATAA	TTGGACTTCACTAATCACGG	86.4	NM_074387
cyp-34A4	TGAACGATTTGAACAGGGTG	TTCTCTGCACATTTCTTTGC	81.4	NM_071696
cyp-34A5	AGTTGTAGAGAAGCTGAGGA	GAACCTCATTAACAACCGCA	98.9	NM_071698
cyp-34A8	AGCTGTTTTTGATAACCGGAA	GGAGCAAATGGAGTAGTTGT	109.3	NM_071701
cyp-34A9	GGCGCAACTATCAATGAAATCC	TCAGCTCTAGCCAGTGATTC	87.6	NM_001047606
cyp-35A2	TTCTGTGCTTTTGGGATACC	TATTACCGTACCTCTTTCTA	79.6	NM_072479
cyp-35A3	CGCTGCGTGTTTAAGTTGGC	TGTTGCCGTATTTCTTTCTG	85.6	NM_071720
cyp-35A4	TCGGCAATTTTGAGTTGGTT	TGTTGCCATATCTCTTTCTA	107.7	NM_071723
cyp-35C1	GAGCCGAGCTGTATTTAATC	ATGTCGAAGGCTTCGCATCT	97.4	NM_171550
cyp-36A1	GATCGATTCTTGAATAGTCGTG	TCGTGATGCTCGGACTGTAA	79.8	NM_059866
cyp-37B1	ATGTTTGAAGGCCACGACAC	TCCGGGTTGTACTGATTTGG	89.8	NM_074708
cyp-42A1	TCTACAACAGGCACGGAAGG	TGTGTCGTGGCCTTCAAATG	122.2	NM_075287
cyp-43A1	AGTCTGCTCGGATTCTTTTT	GTGCCGTAATTTCCTCATAG	82.5	NM_076560
cyp-44A1	ATCGGGAACATTGGGTATTT	CAGTTTGAACATCAGCAGGA	81.5	NM_062651
dyf-7	GTCTGCGTTTCCGTCACAAG	CGGGGAAGCAACAAGTTCTG	124.8	NM_001392826
egl-46	CACCTCAACCGCTTTTCCAAG	ATTTCACATCCGCCTCCTCC	85.1	NM_072293
F56A4.3	ACGAGGGGAATGAATGGCAA	CCATAGGGACCAA-TATCCATGAACT	101.4	NM_071272
K04H4.2	ACGCCGGAATCTGTTGTTCT	CGTTCATTT-GGAAAGGAGGCAT	95.3	NM_001382959
ptr-4	TCCTACCAGACGCGCAATC	GCAACCCATACTGACGGAGT	79.5	NM_076612
T20F5.4	TCATCTACCGAGCAGCCAAC	GAGATGCTCGGTCTCACTGC	94.5	NM_058862
ugt-9	ATGTTGCCCAATAATTTGCT	TGGTTGGAAGACTGTAACAT	100.2	NM_071911
ugt-29	GATGGTGACTAAGGAAATCAAC	CCGATTTTACCAGTGATCCA	102.8	NM_001383270
ugt-45	GAAATTCGGGTTCAGTCACA	TTAATAGCGTCGGTAGTCGG	88	NM_068009
ugt-57	CCTTGTCCGTGAGAAAGTTA	TCCGTCAATCCTTCCGTAT	101.1	NM_076781

[a] Primer efficiency is defined as how many copies of PCR product are produced per cycle. In ideal conditions, the primer efficiency is 100%, meaning that the amount of PCR product is doubled with each cycle. The primer efficiency was used for the calculation of the expression levels.

Table S2. Primer pairs used for qPCR with mouse samples.

Gene name	Forward Primer sequence (5′ – 3′)	Reverse Primer sequence (5′ – 3′)	Efficiency (%)[a]	RefSeq mRNA ID
Ugt3a2	GTGTGTCGCAAGTTCTTCAT	TGATGTTTGGACCTGCCATA	94.7	NM_144845.3
Hpgds	CAGCGTTGGAGCAATGTCAA	CTGCCCAGGTTACATAATTGC	109.9	NM_019455.4
Ugt2a3	TATAGACCCTGTCGTTCCCTGT	TTGTGGGCCTTCCTAGGGTT	92.6	NM_028094.3
Sod2	AACGCCACCGAGGAGAAGTA	TCCAGCAACTCTCCTTTGGGT	102.2	NM_013671.3
Galm	AAGGAACTGCCTTCGACCTG	GACGCACCCTTGCACAAAAC	111.0	NM_176963.4
Gstp1	TGAGTACCCCTCTGTCTACGC	AGCTGCCCATACAGA-CAAGTG	96.4	NM_013541.1
Zfp513	GGGAGGGAGATTCCACAAGC	CCCCTGAGAGTCTCCTTCAGA	146.9	NM_001177901.1
Nfil3	CAGGGAGCAGAACCACGATAAC	CCTCGTCCTACAGACCGGAT	89.9	NM_017373.3
Apod	ATCTGAGAGAAACTGACAG-TGAGC	GAGACGGGCATTTCCCAAGA	108.8	NM_007470.4
Ptchd1	CCCTTTCGTCATGCTAGGTCAT	GCCGAAGGTGACCAAGTACA	123.2	NM_001093750.1

[a] Primer efficiency is defined as how many copies of PCR product are produced per cycle. In ideal conditions, the primer efficiency is 100%, meaning that the amount of PCR product is doubled with each cycle.
